# Do International Health Partnerships contribute to reverse innovation? a mixed methods study of THET-supported partnerships in the UK

**DOI:** 10.1186/s12992-017-0248-2

**Published:** 2017-04-18

**Authors:** Kavian Kulasabanathan, Hamdi Issa, Yasser Bhatti, Matthew Prime, Jacqueline del Castillo, Ara Darzi, Matthew Harris

**Affiliations:** Institute of Global Health Innovation, Imperial College London, 10th Floor, QEQM Building, St Marys Hospital, London, W2 1NY UK

**Keywords:** Reverse innovation, International health partnership, Global health, Development

## Abstract

**Background:**

International health partnerships (IHPs) are changing, with an increased emphasis on mutual accountability and joint agenda setting for both the high- and the low- or middle-income country (LMIC) partners. There is now an important focus on the bi-directionality of learning however for the UK partners, this typically focuses on learning at the individual level, through personal and professional development. We sought to evaluate whether this learning also takes the shape of ‘Reverse Innovation’ –when an idea conceived in a low-income country is subsequently adopted in a higher-income country.

**Methods:**

This mixed methods study used an initial scoping survey of all the UK-leads of the Tropical Health Education Trust (THET)-supported International Health Partnerships (*n* = 114) to ascertain the extent to which the IHPs are or have been vehicles for Reverse Innovation. The survey formed the sampling frame for further deep-dive interviews to focus on volunteers’ experiences and attitudes to learning from LMICs. Interviews of IHP leads (*n* = 12) were audio-recorded and transcribed verbatim. Survey data was analysed descriptively. Interview transcripts were coded thematically, using an inductive approach.

**Results:**

Survey response rate was 27% (*n* = 34). The majority (70%) strongly agreed that supporting LMIC partners best described the mission of the partnership but only 13% of respondents strongly agreed that learning about new innovations and models was a primary mission of their partnership. Although more than half of respondents reported having observed innovative practice in the LMIC, only one IHP respondent indicated that this has led to Reverse Innovation. Interviews with a sample of survey respondents revealed themes primarily around how learning is conceptualised, but also a central power imbalance between the UK and LMIC partners. Paternalistic notions of knowledge could be traced to partnership power dynamics and latent attitudes to LMICs.

**Conclusions:**

Given the global flow of innovation, if High-income countries (HICs) are to benefit from LMIC practices, it is paramount to keep an open mind about where such learning can come from. Making the potential for learning more explicit and facilitating innovation dissemination upon return will ultimately underpin the success of adoption.

## Background

A partner is “a person with a joint share in or use of something” [1]. Yet far from being ‘shared’, International Health Partnerships (IHPs) have long existed as, valuable, and important, but inequitable relationships between a high-income country (HIC) partner and a low- or middle-income country (LMIC) partner. Flow of knowledge, capacity building and service delivery have traditionally been almost exclusively unidirectional.

In the UK, the Tropical Health Education Trust (THET) – a not-for-profit organisation that supports, monitors and evaluates IHPs – itself began its Health Partnership Scheme with the primary objective to ‘improve health outcomes for poor people in the UK Department for International Development (DfID) priority and other low income countries’, focusing on Millennium Development Goals 3, 4 and 5 [2, 3]. However, in the post-development context, the partnership landscape is changing [4]. Increasingly, the narrative has shifted towards shared learning and collaboration. Sustainable Development Goal 17 is dedicated entirely to this concept [5]. Partnerships are shifting further along the ‘spectrum of collaboration’, defined by Rosenberg et al. [6], from short-term, interventionist, ‘coordination’ partnerships through to more integrated, collaborative efforts. This manifests not only as shared agenda setting, for example, as enshrined in the Paris Declaration [7], but crucially also as shared learning.

Within this, there is a recognition that the UK partner should also benefit from the IHP. Focus has almost exclusively centred on learning at the individual-level i.e. personal and professional development (PPD) [8, 9]. Even the most recent initiative to optimise UK partner learning from international volunteering – the Continuing Professional Development (CPD) Toolkit developed by Health Education England [10] – focuses almost solely on PPD. Similarly, recent THET surveys of returning IHP volunteers indicate that the predominant benefit to the UK partner is that of individual development [11].

Given that most UK IHPs between the NHS and overseas institutions, are funded under the Health Partnership Scheme - a DfID funded, and therefore taxpayer-funded programme,- there is a growing imperative to better justify the economic case for IHPs, from a UK perspective, particularly in demonstrating value for the UK NHS. More recently, developed health care systems are operating under increasing financial constraints, understanding the individual and organisational benefits of IHPs for the UK partner is valuable for staff involved in the commissioning and management of NHS human resources [2]. There are multiple opportunities to learn from LMICs [8], for example around improved surgical procedures [12], improved long-term outcomes in mental illness [13–15], and improved skill mix with scaled use of community health workers [16–18]. LMICs need to do more with less, and have done so by integrating public health and clinical medicine, ensuring that patient empowerment is central to health policies and developing health workers that are trained to meet local needs, and not just the needs of professions [8, 19]. However, the literature exhibits limited examples of where a HIC has adopted an innovation from a LMIC, that was conceived and developed by the LMIC [20]. Even where HIC and LMIC institutions collaborate there have been few, if any, explicit examples of this kind of learning – the learning and exchange of expertise is predominantly directed from the HICs towards the LMICs [21–24].

We considered whether, in addition to the clear personal and professional benefits to the individual volunteers, there is scope for additional ‘institutional-level’ learning arising from IHPs, with focus on potential organisational and service delivery changes on the UK side, through observed examples of tangible innovative practices in the LMIC – termed Reverse Innovation. Reverse Innovation refers to an idea, conceived and/or developed in institutions in a lower income setting, that is subsequently adopted to fulfil an unmet need in a higher income setting [25]. The term, originally used by Govindarajan and Trimble [26, 27] was coined in the business world. However, its assimilation into the global health lexicon is associated with the transfer of a practice (e.g. care model, procedure or technology) from a low to high income setting, a transfer that produces the same or better outcomes, for a lower cost – ultimately, more efficient. Whilst connotations of the term have sparked discussion [28] it is used here in line with the body of literature this work is rooted in.

DePasse and Lee [25] observe several incentives in LMICs that lead to the development of innovations. First there is the need for higher volume at lower price. Second, there is often a clean slate to introduce new models of care. Third, that there is pressure for sustainability. Fourth, fewer regulations allow for faster development of innovation. They posit that learning from a LMIC requires a particular type of ‘cross-over’ between the early adopters of the LMIC to innovators in the HIC. HIC innovators need to form spannable social distances with LMIC early adoptors and they propose some strategies to support this process, such as raising the visibility of LMIC innovations, making seed funding available for early innovators in HICs to consider the LMIC innovations, or to create Reverse Innovation zones where ‘outside’ ideas may be efficiently pilot-tested [25]. However, the ‘cross-over’ itself has not yet been adequately explored and, as proposed by DePasse and Lee [25] to support Reverse Innovation, there are few localities explicitly established to pilot and test these innovations in HICs.

Although there has been some discourse to this end, with allusions to institutional learning from innovative practice in LMICs through IHPs [2, 3], there is a paucity of empirical evidence. Many different avenues may exist for ideas and innovations originating in developing countries to be adopted in developed countries. As conversations move towards demonstrating impact and benefit of IHPs upon both partners - IHPs might be a possible avenue for reverse innovation. The UK NHS continues to search for different sources of innovations. Although the concept of reverse innovation is not currently a great priority for current IHPs, it could prove beneficial to the NHS if volunteers were open to learning and capturing ideas and innovations taking place in developing countries. It is not known whether IHPs are effective vehicles for Reverse Innovation. This study set out to identify the extent to which IHPs have led to Reverse Innovation i.e. from observation of innovative practice in the LMIC through to changes in practice in the UK NHS, and to examine the enablers and barriers to the process.

We approached this through a mixed methods study, starting with a scoping survey of all THET supported IHPs and with follow-up interviews of a sample of the UK leads of the partnerships. Note that for the purposes of this study we distinguish between *individual* (i.e. personal or professional development) and *institutional* (i.e. adoption of new procedures, technologies and/or care models) learning, the latter is more in line with our research focus of Reverse Innovation.

## Methods

### Survey

All of the leads of UK THET-affiliated international health partnerships were eligible for participation in the scoping survey. Participants were drawn from the THET database of all past and present partnerships, representing a range of both clinical and managerial cadres. We exclusively looked at UK partners, which in general, are NHS hospitals or higher education institutions or academic health science centres and that serve as the HIC partner to which RI would flow. No representatives from the LMIC partners were contacted in the study. The survey, which was web-based, focused on the domain of learning with a view to capture institutional changes, rather than personal and professional development at an individual level. Following initial drafting, to ensure face validity, ease of use and relevance to the research question, the survey was piloted and revised through critical multidisciplinary review from the THET Evaluation team and the Innovation Research group at Institute of Global Health Innovation, Imperial College London, which includes designers, surgeons, sociologists and clinicians.

The survey comprised two broad sections. The first focused on the characteristics and objectives of the partnership, through a series of multiple-choice questions. The second explored examples of innovative LMIC procedures, technologies and/or care models that had been observed as part of the overseas placements with the LMIC institution and whether any attempts were made to share learning of these innovative practices in the UK institution. Respondents were also invited to provide contact details for follow-up interviews. The survey was distributed by email on the 7^th^ March 2016 with two reminders sent over the following fortnight. In order to ensure confidentiality and anonymity, and because they are responsible for ensuring monitoring and evaluation of the IHPs, the THET Evaluation team distributed the survey. The researchers had no access to any respondent contact information unless provided voluntarily by them at the end of the survey.

### Interviews

Informants were drawn from the pool of initial survey respondents who had indicated at the end of the survey that they would be prepared to participate in a follow-up interview and had provided their contact details voluntarily. Times and dates were scheduled at their convenience, over the course of April through to early May 2016. Interviews were semi-structured, led by a brief topic guide facilitating flexible exploration of contrasting themes that emerged. The overarching exploratory focus was the enablers and barriers to learning from LMIC partners through IHPs, the discussion were loosely shaped by four pre-determined topic areas: considering experiences of learning of the informant, the role of the partnership itself in such learning, the role of other actors and finally suggestions to maximise and optimise learning.

**Fig. 1:**
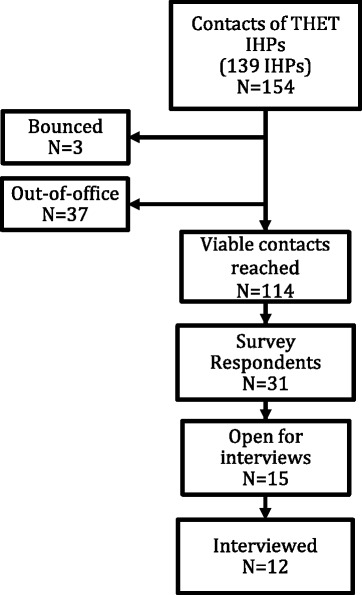
Recruitment flow chart for survey and interviews

Interviews were conducted over the telephone by one researcher (KK), and lasted between 30 and 60 min. All interviews were audio-recorded and subsequently verbatim transcribed, the first four by the primary researcher (KK) and the remainder by a commercial medical transcription company, owing to time constraints. Strict confidentiality was maintained throughout, with removal of identifiable information upon receipt of transcripts. A separate informant list, with corresponding reference code was kept under password-protected file. We obtained oral informed consent from all informants, with freedom to withdraw this consent at any time, although this was not exercised by any of the respondents (Fig. 1).

#### Analysis

##### Survey

Respondent characteristics and multiple-choice questions on the partnership objectives and characteristics were analysed descriptively. Baseline characteristics for the THET-affiliated partnership population were provided by THET from their database.

##### Interviews

Interviews were analysed inductively through a thematic analysis [29]. Four transcripts were initially selected and independently reviewed by two researchers (KK and MH). By analysing informant responses on both a semantic and latent level [30], themes were identified within and across transcripts, which was reflected in a maiden code structure with major and minor codes. The two researchers re-reviewed the first four transcripts with the maiden code structure and revised these early codes recursively by modifying, amalgamating and retiring codes where necessary [31, 32]. The code structure was then re-applied to the first four transcripts, repeating the process until both researchers reached agreement that the codes captured shared themes within and across all four transcripts. The resulting code structure was applied to all transcripts by one researcher collating subunits of text relevant to each code. To ensure codes were correctly applied to the data text subunits and collated extracts were reviewed by the second researcher. Disputes were settled through discussion and consensus. The final codes, informed by corresponding data extracts, formed the basis of theme and sub-theme synthesis, themselves mapped to postulate possible inter-theme explanatory relationships [33]. Interviews generated in excess of 150 pages of single-spaced, 12 pt font transcripts. Coding of all transcripts was conducted manually, given the volume of data and the advantages of complete immersion in the data through this approach.

## Results

### Survey

We received 31 responses from the total pool of viable contacts (*n* = 114, response rate 27.3%). Responder and partnership characteristics are comparable to the base population of THET-affiliated partnerships (Table 1).

**Table 1:** Characteristics of THET partnerships in general and those that participated in the survey

	All thet-affiliated partnerships (*N* = 139)	Respondent partnerships (*N* = 31)
Africa (%)	81.0	83.9
Southeast Asia (%)	16.5	12.9
Eastern Mediterranean (%)	2.2	0
Western Pacific (%)	1.4	0
Europe (%)	0.7	3.2
Americas (%)	0	0
Multi-country partnership (%)	9.4	9.7
Mean duration (MONTHS)	20.8	21.6
Still ongoing (%)	70.5	80.6

Reflective of THET partnerships generally, the majority of respondents represented partnerships with African countries (81%). Three respondents represented multi-country partnerships, which were also the largest partnerships, both in terms of duration (up to 58 months) and funding (up to £600,000 per annum). The mean period of respondent partnerships was 21.6 months, although this includes periods prior to THET support in some instances. Respondents were from a range of cadres, clinical to managerial.

On average, IHPs had twenty-two people directly involved in the partnership from the UK side (range 2 to 223) with the majority being frontline health workers (50%), whilst 14% were academics or researchers and only 7 and 6% managers or senior executives respectively. On average, the partnerships had the involvement of only one senior executive, and 14 of the 31 IHPs that responded had no participation from either a senior executive or a manager.

The majority of respondents (20 of 31) indicated that the most important purpose of their partnership is teaching. Research and advocacy were least likely to be ranked as the most important purpose of the partnership. The majority (77%) strongly agreed that ‘supporting partners’ best described the mission of the partnership. Fewer respondents (55%) strongly agreed that the partnership was about building a reciprocal relationship, and fewer still (12%) strongly agreed that a purpose of the partnership was to learn about new models of care or innovations from the LMIC partners.

The survey also explored tangible innovations (e.g. new procedures, technologies and care models) that the respondent had seen during their partnership, probing the extent to which these observations led to any change in practice in the UK. Although over half of respondents (16 of 31) reported seeing an innovation in the LMIC, the number who endeavoured to share these on return, however, was just over a half of that, and only one IHP respondent indicated that a Reverse Innovation is taking place.

### Interviews

Although respondents were a self-selecting sub-sample of survey respondents, interviews were an opportunity to probe deeper into the experiences from the LMIC partnership and the type of learning that had taken place from the perspective of the UK volunteers. Particular emphasis was given to understand how partnerships are reciprocal and what constitutes reciprocity.

The respondents were the leads of the partnership, and were speaking in part on behalf of the many volunteers that had been involved in the partnership, but speaking also from their own experiences as volunteers in the past, or current, within the partnership. As leads for the partnership, the respondents were in positions of responsibility to know and understand the experiences of their volunteers through debriefing post-visit, and also through partnership evaluations, often initiated at the request of THET as a condition of the partnership support.

Two main themes emerged from the interviews – first, the manner in which learning was referred to had little to do with ‘institutional learning’; second, that perceptible ‘West is Best’ narratives are rooted in a moral imperative to help rather than be helped, and that this undermines to some extent the possibility to learn from innovative practices in the LMIC. An obvious conflicting tension exists between the narrative of reciprocity and the moral imperative to assist LMIC partners. We found at times some respondents embodied a form of *‘pseudo-empowerment’* as they manoeuvred through the practicalities of this tension.

### Learning, but not learning; reciprocal, but not reciprocal

We asked informants what was learnt from their experiences in LMICs. This took many forms but almost none was to do with the ‘institutional’ type that we conceptualised as Reverse Innovation.

There was a great appreciation of the need to learn from the experience –

*“Within our peer group at the end of each year it’s always about what did you learn, what did you gain from staff who have been out there as much as what you have achieved in terms of changes to health care in our link country. This is equally fully discussed.”* 26^th^ April 2016, Senior Manager, African partnership

However, when asked what was learnt from the partnership, most notable was a distinct emphasis on personal learning, with particular focus on professional development but also learning how to teach better, and learning how to do better international development. It surprised us to find that none of these approaches to learning seem to fit well with the notion of ‘how to improve care in the UK’, or more precisely ‘how to apply LMIC care models in the UK’, particularly given a prevailing narrative around IHPs as reciprocal, mutually beneficial arrangements between the UK and LMICs [1–6].

Professional development was commonly reported, specifically the sharpening of clinical skills:

*“For [doctors on the ground] a major learning curve was to really start to use the clinical skills…hone in on the clinical signs and use their clinical ability to make decisions”* 21^st^ April 2016, Senior Academic Administrator, African partnership lead

*“…having to make clinical decisions without necessarily having the back-up of the same equipment…”* 27^th^ April 2016, Anaesthetist, African partnership

Equally, the partnerships were viewed as valued opportunities for volunteers to become more culturally aware:

*“there comes an awareness of diversity through health partnerships, of the sort that comes from working with people from a completely different culture to yourself”* 21^st^ April 2016, Senior Academic Nurse, African partnership lead

Spending time in the resource-constrained partner settings made volunteers more ‘resourceful’, although respondents did not provide examples of how this has changed their practice practically speaking once they returned to the UK. The partnership, and working in the LMIC environment leads to observation and reflection, giving volunteer’s time and space to look in from the outside, to see things in context, and compare them to their own practice.

*“…for them to work in an environment which was so short of resources, yet making things work… you tend to moan about things not being adequate here, but when you work in that kind of environment, you can’t help but ask yourself ‘what’s our problem?’…we have none. They [UK partners] can only be more positive about how things are here.”* 11^th^ April 2016, Retired Public Health consultant, African partnership lead

*“That if you watch other people being resourceful, it does make you realize that here we just take so much for granted. And we complain about all sorts of nonsense. When just a little bit of resourcefulness or innovation, on a day to day practical level, would enable us to manage”* 15^th^ April 2016, Plastic and Reconstructive surgeon, African partnership lead

Another very clear and frequent theme was that the volunteer experience was an opportunity to improve upon their own teaching practice, in the LMIC context. Lessons learnt were more about teaching style and the best way of delivering teaching to the LMIC partners:

*“What lessons did we learn from India? Well…the important thing is that the people you’re training have to see the benefits of it, to them but also to the people that they’re looking after. So, we found that scenario based training, or case studies were far better than lecture presentations, so we were looking at small group work. We learnt this after the first round of training, so we learnt from experience and adapting the training.”* 9^th^ April 2016, Clinician, South-Asian partnership lead

Finally, doing better international development through better teaching, being open to the needs of LMIC partners, engaging them in their own development and their own learning process was frequently noted as an important element of what is learnt from the partnership:

*“So this is very much a partnership of not one being more skilled than the other but one being able to adapt to help and support the low income country professionals to incorporate some of the new technologies within the limitations of their resources. So it’s always been clear to us that learning is two way”* 26^th^ April 2016, Senior Manager, African partnership

We found it noteworthy that our respondents were able to easily detail the benefits to the individual volunteer, from improving clinical skills, to improving teaching techniques and understanding how to be better partners in the international development process. Equally, we found it significant that the respondents considered these benefits to be evidence of a reciprocal relationship with the LMIC partner. In effect, ‘it is reciprocal because we get something out of it’ - but yet ultimately not in the sense of learning from the LMIC partners and how they ‘do things’, and how might these provide lessons for the NHS.

### “We come from a system that works” – latent power imbalances in health partnerships

The claim of reciprocity in IHPs seems to be limited to issues of personal and professional development for the UK volunteers. Individual volunteers learn a lot, for themselves predominantly, on issues of clinical skills, and teaching and generally how to ‘do’ international development better. This seems to constitute that which is held to be reciprocal about the partnership. This, to us, is at odds with a broader understanding of reciprocity– i.e. to explore in equal measure what could be learnt from the LMIC health system, as much as that which could be taught.

The structures and processes of the health partnership seem to play a role in this. The two most noticeable factors that arose repeatedly throughout the interviews were those of funding direction and locational disparities. The activity of the health partnership is almost exclusively based in the LMIC. Were IHPs to be as truly bilateral, or bidirectional, as they aspire to be, then perhaps activities would be co-located. Volunteers, however, almost exclusively travel to the LMIC, and funding is predominantly from the UK Government. This, perhaps inevitably, sets an agenda:

*“The money is British taxpayer’s money. So you can argue to your heart’s content that it might be better to [bring neighbouring African doctors in to help] but you can’t use THET’s money and say to THET, ‘I’m taking your money but we’re not going to do anything you wanted us to.*” 18^th^ April 2016, Orthopaedic surgeon, African partnership lead

From the respondents in our sample, there were vanishingly few examples of activity where the LMIC partner visited the UK, and strikingly, where a visit did take place this seemed to provide sufficient basis to claim that the partnership exhibited shared learning and collaboration:

*“we’ve got a man coming over from India, MK…in May to deliver to a series of lectures to UK, for the price of an airfare. So very much we see this as shared learning and collaboration, it’s not one way, it’s as much two way”* 9^th^ April 2016, Clinician, South-Asian partnership lead

In addition to how IHPs are organised and delivered, a significant theme arising from the interviews focused on the intrinsic motivation for involvement in the partnership. Underpinning our observation that learning is predominantly clinical, or managerial, or learning to do international development better, was a deeper sense of a moral and ethical duty to help and support the LMIC partners in their own development. We do not place any value judgment on the appropriateness, or otherwise, of this ethical standpoint, other than to observe that this seems also to constrain respondents with respect to ‘institutional learning’. Not only might it establish a dependency-type relationship with the LMIC (and much has been discussed in this regard in the development literature [34–36], equally likely (and less discussed) is that it also fosters an anti-learning agenda. If one is preoccupied with the task of teaching (selflessly) then it is hard to see opportunities also to learn.

*“…there’s those who judge the whole partnership on what they learn…which is…quite a negative way of looking at such a partnership. Because you haven’t gone into this partnership to see what you can get out of it – you’ve gone into this partnership because there’s a big clinical need for training, or for clinical services, and you want to be part of the partnership that delivers for that need.”* 18^th^ April 2016, Orthopaedic surgeon, African partnership lead

*“All our resources had to be concentrated on Malawi because I think that was the ethical thing to do.”* 21^st^ April 2016, Senior Academic Nurse, African partnership lead

This intrinsic motivation to help and support the LMIC country is furthered by the belief that the UK health system works, and there is a role, if not duty, for UK volunteers to share that knowledge:

*“The first few [IHP projects] that they go on, [UK partners] come with an agenda, not of superiority, but with an agenda of ‘we come from a system that works – we’re going to share what we know with a system that doesn’t work’. So less inclined…so less open to picking things up, more wanting to share what they’ve got.”* 18^th^ April 2016, Orthopaedic surgeon, African partnership lead

From our interviews, we noted a striking ascension of the UK partner to the role of primary knowledge broker in the health partnership – that is, the holder of expertise and know-how as to the ‘correct’ way of doing things. For the overwhelming majority of informants, there was a tacit assumption that they *should* be providing an intervention:

*“we were quite worried that we were just sending lots of people who just go and look around and don’t actually do anything”* 27^th^ April 2016, Anaesthetist, African partnership

### The ‘work-around’ – an issue of pseudo-empowerment

Some informants revealed an apparent, intriguing work-around to the conflicting tensions of reciprocity and the moral imperative to assist the LMIC partner, which we refer to as a form of *‘pseudo-empowerment’*:

“*…I often do try and say in a way we actually learn a huge amount from you as well…… I think it empowers them…”* 27^th^ April 2016, Anaesthetist, African partnership

By stating that a lot has been learnt, then the LMIC partner might *feel* empowered. Rather than engage in convoluted mechanisms to *appear* to learn, in an attempt to dispel any notion that the UK partners were there for one thing only i.e. to teach, we wonder whether it is simpler (and better) just to be *genuine* about learning. Such is the drive to help, that little attention is paid to the possibility of learning beyond the individual level. We reflect that perhaps a truly mutual learning agenda would, in fact, have been supported precisely by ‘looking around and *not* doing anything’. West is not Best, and neither is the South; there is only need and opportunity, wherever that may lie. The way that IHPs are currently configured seems to shape the learning agenda in a certain way, compromising the concern to learn, meaningfully, from their LMIC partners. Health partnerships do not have the self-interest motive to the same extent as a trade partnerships. However, if IHPs are to be considered truly reciprocal, then the issue of institutional learning may need to be addressed.

## Discussion

We set out to understand whether IHPs are vehicles for Reverse Innovation, the observation of and subsequent adoption of innovative, useful practices from LMICs. We found that although respondents have observed some innovative practices in LMICs, factors such as power imbalances within partnerships and latent attitudes to LMICs appear to constrain their motivation to learn from LMICs in any way other than to improve their own personal clinical practice, teaching practice or international development practice. IHPs are currently fundamentally about supporting the LMIC partners, and volunteers are fundamentally driven to teach and support the partners, rather than learn from them. The North–south directionality is therefore retained, and the UK partners remain in a position of relative power, in part due to the funding of the partnerships.

This is problematic, if IHPs are to claim that they are reciprocal, and mutually beneficial in a meaningful sense. This is not a mere semantic issue. There are significant opportunities to learn from LMICs, and there is a significant need for the NHS to learn from novel approaches to healthcare from diverse contexts. If IHPs are to be a conduit for this type of learning, and if IHPs are to be genuinely, not rhetorically, reciprocal, then the power imbalance in the IHP needs to be redressed.

The past two decades have seen a paradigm shift in the development arena from conditionality-driven, aid-dominated North–south relationships, embodied by the Structural Adjustment Programmes of the nineties, to a greater emphasis on partnership and lower-income country agenda setting input. However, there are extensive critiques of this change [37–39], in particular questioning the extent to which these newly-styled ‘partnerships’ place both the high and lower income countries on an equal footing. Whilst there is overt importance placed upon the idea of recipient ‘ownership’, with the World Bank [40] stating ‘the need to put committed developing country governments and their people at the centre of their development process’, even the more ardent defenders [41] of this shift towards ‘partnerships’ have highlighted the inherent challenge.

As Abrahamsen [42] puts it, ‘one party is in possession of the purse and the other the begging bowl’ (p. 1454). The assertion such partnerships are nothing more than ‘western wol[ves] in African Sheepskin[s]’ [43] may only capture the strongest sentiments against the claims of a genuine change in attitudes, but fears around its use in covert preservation of a neo-colonial world order certainly exist amongst a swathe of commentators [38, 44, 45]. Fowler [46] posits different drivers for the shift towards partnerships – the most controversial of these suggesting them to be a ‘more subtle form of external power imposition’ (p.7), playing the instrumental role of a ‘Trojan horse’ (p.7), a sentiment echoed by Crawford [37].

Crawford [37] goes so far as to suggest the very structure of key governing bodies within partnerships, taking the example of Indonesia, is set-up to protect the status-quo of HIC dominance. Maxwell and Riddell [47] proposed a continuum for partnerships. At one end there are ‘strong’ partnerships, where there is truly shared agenda setting and contractually-binding financial agreements that render the relationship almost transactional. At the other end, there are ‘weak’ partnerships, where there is little more than a limited degree of shared information and discussion about policies. They argue that the majority of international development partnerships still operate within the realms of the latter.

We highlighted a tension in our data – the difficultly in not appearing imposing whilst at the same time getting the job done of training LMIC partners, and improving services. The reason for this incongruence between actions and words is likely not straightforward. We tentatively suggest that this is the result of a tension between the global narrative of collaboration and co-development [48, 49] and an intent to improve clinical practice in LMICs rooted in the belief that the UK system is superior.

Unfortunately a comprehensive analysis of power dynamics, and how they impact upon learning, is beyond the scope of this paper [50, 51]. However, it may be helpful to explore this dynamic through that lens moving forward. Abrahamsen et al. [42] purport that under the guise of local ownership of programmes, and engagement of LMICs as stakeholders, HIC are able to exercise a form of indirect governance over the LMIC through IHPs. Barnett and Duval [52] propose four conceptualisations of power, partly mapping to Lukes’ original three dimensions [53]. The findings from our research may be located within the fourth, and most abstract of the domains, that of ‘productive power’ [52]– this ‘concerns discourse, the social processes and the systems of knowledge through which meaning is produced, fixed, lived, experienced, and transformed’ (p. 55). Our respondents’ experiences seem to indicate that a very specific and entrenched learning dynamic is shaped by what constitutes ‘knowledge’, who is ‘expert’ and where the ‘need’ is.

THET [54–56] emphasises the primary objectives of IHPs as service delivery and LMIC capacity building. Although this has moral and ethical weight, it would be even more ennobled if a genuine ‘institutional learning’ agenda, such as one that incorporates Reverse Innovation, was integrated into its programmes.

Increasingly, the global narrative of IHPs is about engaging partners and creating solutions that work for them, whilst also learning from them. This narrative is captured in THET’s definition of a partnership as *‘a model for improving health and health services based on ideas of co-development between actors and institutions from different countries. The partnerships are long-term but not permanent and are based on ideas of reciprocal learning and mutual benefits.*’ (THET, In our mutual interest). The notion of reverse innovation in its broadest sense is concerned with developed countries adopting specific practices or technology originating in developing countries. To reach such a stage there has to be a mutually beneficial space that encourages partners to exchange ideas and experiences. It is only through this exchange that the process of reverse innovation will become feasible.

However, this learning must extend beyond individual clinical, teaching and international development practice for the UK volunteers. And it must extend beyond a rhetorical nod in the direction of ‘learning’. In previous research we have shown that innovations from LMICs might be discounted early on [57], in part because these contexts are considered to be too different for any learning to be relevant, but also because of some entrenched preconceptions about whether they would have anything to offer a HIC health system [28]. This can translate into measurable bias even in evidence interpretation in some instances [58]. If we are to keep pace with the changing global health landscape, then it is necessary to actively question, and update, the motivation, and practices that underpin IHPs. The role assumed by the LMIC partner, and their expectations, also warrants further scrutiny as it may be exacerbating the power differential.

The figure below compares and contrasts current and potential roles of THET, IHPs and volunteers for a more conducive landscape that supports Reverse Innovation (Table 2).

**Table 2:** Current and potential roles of THET, IHPs and UK volunteers

	Current role	Potential role
THET	Coordinate international health partnerships to improve health outcomes for developing country partner.	Coordinate international health partnerships to drive shared learning that improves health outcomes for both developed and developing country partners.
International health partnerships	Partner with a developing country to support and strengthen the developing country healthcare system.	Partner with developing country to support and strengthen the developing country healthcare system whilst finding ways to support developed health system as well.
UK volunteers	UK volunteers go overseas to teach and share their skills, expertise and models of practice with the developing country partner.	Whilst teaching and sharing skills and expertise – learn about the different ideas and models of practice taking place in the developing country.

Some potential recommendations include to incorporate explicit objectives to observe and learn from LMIC practice into the IHP mission statements; to establish formal forums to disseminate observed LMIC practice within the NHS organisations; to ensure a greater variety of volunteers within the IHPs, including senior managers that can span ‘social distances’ [24, 59], central to the diffusion of innovations; and to ensure that the IHP activity is spent as much in the UK as it is in the LMIC. The recent THET report ‘In Our Mutual Interest’ signals an important shift towards bidirectional learning and knowledge flow that may become embedded in partnership activity moving forward [60].

### Limitations

The objective of the study was to scope the role of IHPs as vehicles for Reverse Innovation, and examine in more detail the personal experiences of IHPs among some respondents. As research based on survey data and in-depth interviews, the study is subject to the inevitable issues of selection bias and recall bias. Further, our respondents might not be representative of the IHP leads as a whole, although the respondents were drawn from the whole universe of THET-supported IHPs. These were a self-selecting group of individuals that were motivated to provide more insights and detail into the IHPs and the learning process and they were drawn from another self-selecting group of respondents that participated in the on-line scoping survey. However, to ensure that our findings and interpretation of the data were relevant and accurate, we discussed them in detail with the THET leadership, and the Monitoring and Evaluation Team for IHPs, which aided the generation of further insights and critique of the research. We have little reason to believe that the data is not representative of the broader shared experiences, mindful that there may be other experiences that could have been described. Further, the study was cross-sectional and largely retrospective in nature, capturing dynamics and nuances of the projects at one snapshot in time, rather than longitudinally. The analytical approach involved manual coding of the data that enabled complete immersion into the data, and the second reviewer was able to check for inconsistencies in collating relevant text subunits. The study may be further limited by varied understanding of what constitutes an innovation. This is a notoriously difficult issue because the word ‘innovation’ can mean many things to many people. We opted for a broad definition to capture the experiences of the respondents and described it as any ‘practices in the LMIC that you have not encountered before, and/or that provide equal or better outcomes at a lower cost than compared to in the UK?’ We also added that ‘these practices may include new procedures, technologies or care models’. We did not specifically explore the nuance of the understanding of the term, because we were necessarily more focussed on understanding the issues around bringing knowledge back to the UK.

## Conclusion

With increasing globalisation comes increasing global innovation flow – and HICs have an imperative to be open to learning from unexpected sources. Our examination of IHPs has illustrated that volunteers work within a web of expectation that learning is not only limited to soft skills, but that LMICs are unlikely to have much to offer in the way of new models of care. Other than a rhetorical narrative for IHPs to be mutually beneficial, they are not currently well supported to truly be so. A combination of insufficient emphasis on institutional learning, geographic LMIC-centricity and subtle preconceptions about LMIC knowledge brokerage seem to be suffocating underlying inclination for pursuit of Reverse Innovation. Attention to these extrinsic structures and processes may serve to reframe the picture to be more balanced and may eventually redress the long-standing attitude of ‘West is Best’.
